# The neurodevelopment of delay discounting for monetary rewards in pre-adolescent children

**DOI:** 10.1038/s41598-021-87282-z

**Published:** 2021-04-16

**Authors:** Mei Yu, Tongran Liu, Fangfang Shangguan, Jingxin Sui, Jiannong Shi

**Affiliations:** 1 CAS Key Laboratory of Behavioral Science, Institute of Psychology, Chinese Academy of Sciences, 16 Lincui Road, Chaoyang District, Beijing, 100101 China; 2 Department of Psychology, University of Chinese Academy of Sciences, Beijing, 100049 China; 3 Beijing Key Laboratory of Learning and Cognition, School of Psychology, Capital Normal University, Beijing, China; 4 Student Office, Liaoning Normal University, Dalian, 116029 Liaoning China; 5 Department of Learning and Philosophy, Aalborg University, Aalborg, Denmark

**Keywords:** Cognitive neuroscience, Human behaviour

## Abstract

Children are found to exhibit high degrees of delay discounting compared with adults in many delay discounting studies, which might be due to the asynchronous development of “bottom-up” and “top-down” neural systems. However, the temporal dynamics associated with the two systems in the development of delay discounting processes are not well known. In this study, we chose two age groups of participants and adopted event-related potential (ERP) techniques to investigate the neural dynamic differences between children and adults during delay discounting processes. Behavioral findings showed that children discounted more than adults and chose more immediate choices. Electrophysiological findings revealed that children exhibited longer neural processing (longer P2 latency) than adults during the early detection and identification phase. Children showed less cognitive control (smaller N2 amplitude) than adults over the middle frontal areas, and they devoted more neural effort (larger P3 amplitudes) to making final choices than adults. The factors of reward amount and time delay could influence the development of delay discounting in children.

## Introduction

Pre-adolescent children are often characterized as impulsive decision makers and frequently ignore the long-term benefit of their choices. When making a choice between a smaller and sooner option and a larger and longer option, children aged 6–11 years old are more likely to be driven by reward immediacy, while adolescents aged 12–17 years old are more likely to be driven by reward amount^1^. It can be assumed that adults may also be driven mainly by reward amount, and children may choose smaller and sooner alternatives more than adults as a prior study found^2^. For example, when facing the choice of receiving 10 dollars now or obtaining 20 dollars after a month, more children than adults will choose the option of receiving 10 dollars now. The result of differently weighting the two options leads to the subjective value of 10 dollars now being higher than 20 dollars after a month, even though the objective value is inverse. This kind of choice, referring to the decrease in a reward’s subjective value as the delay to its receipt increases, is called delay discounting^3–11^.

In the widely used paradigm of studying delay discounting^12^, the immediate choices varied in terms of the amount (from $0.01 to $10.50) and were received immediately, whereas the delayed choices varied in terms of the time delay (from 0 to 365 days), but the amount was fixed at $10. The immediate choices and delayed choices were paired in advance but were presented to participants randomly. The delay discounting phenomenon is often described by the hyperbolic model from Mazur^13^ (see Appendix in the Supplementary file). In the model, a larger k value indicates that the person’s degree of discounting for the delayed reward increases more rapidly^14^, and the person is more likely to choose immediate choices. Mitchell et al.’s paradigm^12^ was widely used in children^15,16^ and adults^17,18^, and its adaption was used in event-related potential (ERP)^19^ and functional magnetic resonance imaging (fMRI) methods^20^.

Moreover, it has been found that children’s discounting rate was higher than that of adults in many developmental studies^2,21–23^. However, the neural mechanisms for the neurodevelopment of delay discounting processes in children have been less studied. During immediate reward selection in delay discounting, it was found that some brain areas were significantly activated such as ventral striatum, medial orbitofrontal cortex (MOFC), medial prefrontal cortex (MPFC), posterior cingulate cortex (PCC), and posterior hippocampus^24^, which were related to “bottom-up” system, motivated by rewards and related to reward circuitry^25^. Meanwhile, during choice making period, some brain areas were significantly activated such as areas of the intraparietal cortex, dorsolateral prefrontal cortex (DLPFC), ventrolateral prefrontal cortex (VLPFC), and lateral orbitofrontal cortex (LOFC)^24^, which were involved in “top-down” system that regulated impulsive behaviors and related to cognitive control areas^26,27^. In delay discounting, the relative engagement of the two neural systems is associated with one’s choices, with greater relative fronto-parietal activity when one chooses delayed rewards^24^. During human development, the two systems mature asynchronously, which influences children's decision makings^27–31^. It is believed that the “bottom-up” system develops faster than “top-down” system^32^, which might lead children to focus more on the immediate choices in delay discounting.

In a fMRI study, Christakou et al.^33^ tested the development of delay discounting in a sample of individuals between 12 and 31 years old and found a linear decrease in discounting with age. It was further discovered that the ventromedial prefrontal cortex (VMPFC) contributed to the development of self-control by connecting delay-related information from brain areas such as the DLPFC, insula, and parietal cortex. Moreover, de Water et al.^20^ found that adolescents aged 12–16 years old showed a close connection between delay sensitivity and the neural activation of cognitive control processes, and they also observed a close relationship between amount sensitivity and the neural activation of reward valuation^20^. Meanwhile, researchers recruited adolescents aged 14–16 years old and adults aged 18–23 years old to complete a valuation task during which subjects’ brain electrical activities were recorded during a delay discounting task, and found that adolescents devalued delayed rewards more than adults which could explain adolescents’ higher discounting^34^. However, few studies have paid much attention to the characteristics of children’s temporal dynamics in delay discounting.

Several ERP components are related to the time course of delay discounting processes. The frontal P2 component is related to the task-relevance of stimulus items^35^ and involved in the “bottom-up” system. It occurs approximately 150–200 ms poststimulus onset and is a good index for the early detection and identification of task-related perceptual representations^36^. It was discovered that P2 amplitudes varied as the time delay increased from 2 weeks to 50 years^37^. P2 responses were larger with long delays than with short delays and larger with large amount than with small amount^19^. It was also found that in delay discounting task adults preferring immediate rewards showed delayed P2 responses^38^. P2 amplitudes increased and latencies decreased with development in children aged 6–13 years old during an oddball task^39^.

Two ERP components are related to cognitive control processes and refer to the “top-down” system. The first one is the frontal N2 component, which is often referred to in decision making, and it often appears between 200 and 350 ms after stimulus onset and is involved in cognitive control processes, such as cognitive flexibility and inhibitory control^40–42^. Neural activations in cognitive control areas were associated with delayed choices, and larger N2 amplitudes were related to nonimpulsive choices; thus, N2 may act as a neural marker for one’s ability to resist immediate rewards in delay discounting^19,24^. Children aged 7 years old with more activation of the DLPFC showed more negative N2 amplitudes in response to nogo trials in a go/nogo task, indicating that more cognitive control resources were recruited during response inhibition^43^. The second one is the parietal P3, which is a positive and large-amplitude ERP component with a typical peak latency between 300 and 400 ms over central-parietal areas^44^. This component reflects the response to stimulus evaluation and decision making^44^ and is also thought to be related to the processing capacity and mental workload^45^. During delay discounting processes, P3 amplitudes were significantly larger in short-term delays (involving more nonimpulsive decisions) than in long-term delays^19^. Earthquake survivors who exhibited larger P3 amplitudes compared with normal adults were regarded as being more impulsive and having less mature cognitive control in a monetary delay discounting task^46,47^. Children’s preference for immediate rewards may be due to their immature cognitive control and their willingness to engage in high-risk behavior; more evidence is needed to investigate the relationships among children’s P3 responses, cognitive control and delay discounting.

It should be noted that during one’s development, the right and left brain hemispheres have different speed of development. It was found that neonatal babies born about 40 weeks exhibited greater brain network efficiency in the left hemisphere compared with the right hemisphere^48^. Besides, a prior study recruiting subjects aged from 4 to 21 years old found that the prefrontal and inferior parietal cortices on the left hemisphere matured earlier than those on the right hemisphere, which may be due to most subjects in the study were right-handed and so had left-dominant and right-lag hemisphere^49^. However, findings are not always the same. Suyu et al. found that white matter network in right hemisphere is more efficient than that in left hemisphere in adolescents aged from 11 to 15.9 years old and young adults aged from 21 to 25.9 years old^50^. The differences illustrated that different brain structures had different hemispherical development characteristics. It was found that in adults the enhanced activation of the left hemisphere could promote normative decision making in a ratio bias problem task^51^. Meanwhile, in delay discounting, it was found that the “bottom-up” areas inclined to the left side such as left posterior hippocampus and the “top-down” areas were mainly on the right side such as right DLPFC, right VLPFC, and right LOFC were activated significantly^24^. It is a question that how the developing discrepancy and the different hemisphere activity will influence the temporal dynamics of age differences in delay discounting.

Reward amount and time delay are two basic factors in delay discounting, and they were found to have dissociable neural activities: mesial prefrontal cortical activity was positively correlated with future reward magnitude, and DLPFC and posterior parietal cortical activity (PPC) were negatively correlated with future reward delay^52^. In a prior study with adult subjects, the immediate reward amount was fixed to ¥50 and the delayed reward amount varied in small amount of ¥60 and large amount of ¥100, and the time delay varied from 1 day later to 5 days later as short time delay and from 6 months later to 12 months later as long time delay^19^. It was found that P2 was relatively larger in large amount condition compared with small amount condition and larger in long delay condition compared with short delay condition. N2 and P3 were larger in short delay condition involving lower discounting choices compared with long delay condition^19^. In addition, Scheres et al.^1^ discovered that the choice of children aged between 6 and 11 years old was driven by reward immediacy, while for adolescents aged between 12 and 17 years old, their choices were more influenced by reward amount, which may lead to an increased preference for delayed choices with increasing age^1^. However, the findings of whether reward amount and time delay affected children’s neural dynamic processes of delay discounting differently from adults are less well known and require further exploration.

In the present study, we intended to find out how the asynchronous development of the “bottom-up” system and the “top-down” system influenced children’s performance in delay discounting in the perspective of temporal dynamics. As a previous study found that individuals’ behaviors were significantly predicted by N2 amplitudes in adults^19^, we hoped to discover whether the behaviors of children and adults were predicted by different ERP components reflecting different neural systems. Furthermore, we hoped to reveal how the differences of children and adults in the two neural systems were affected by reward amount and time delay. It’s believed that the “bottom-up” system develops faster than the “top-down” system during one’s development^32^. We hypothesized that the differences between children and adults in P2 component reflecting the “bottom-up” system were smaller than those in N2 and P3 reflecting “top-down” system. Meanwhile, it was hypothesized that children may exhibit smaller and delayed frontal P2, which reflects developmental lag^39^ and immature “bottom-up” development. More neural efforts were needed in children to complete the task by inducing larger P3 responses^53^. It’s also hypothesized that children might show smaller N2 and larger P3, which shows the undeveloped “top-down” system. What’s more, prior studies found that N2 amplitudes might be the key component in delay discounting and could predict adults’ delay discounting behavior, with smaller N2 amplitudes correlated with more immediate choices in delay discounting^19,46^. Due to children’s undeveloped neural systems, we hypothesized that the components that might predict children’s choices would be different. In addition, as reward amount and time delay influenced one’s delay discounting behavior^54^, we predicted that children and adults would be influenced by reward amount and time delay differently. Since children’s discounting behaviors were mainly driven by reward immediacy, while older individuals’ discounting behaviors were mainly motivated by reward amount^1^, the differences of two neural systems between adults and children might be mainly in one dimension of reward amount or in one dimension of time delay. In the current study, we adopted a potential real reward design, which was regarded as suitable for children to make every choice as if it was real^11,23^ since children’s experiences with money and time are limited. As previous studies found that delay discounting was influenced by individuals’ intelligence^2,55^ and impulsivity^56^, we used Raven’s Standard Progressive Matrices (RSPM)^57^ to assess participants’ intelligence and a Chinese revised version of Barratt Impulsiveness Scale (Version 11)^58^ to measure individuals’ impulsivity in order to reveal whether these factors have influences on the age differences in temporal dynamics of delay discounting.

## Results

### Behavioral results

The Raven scores of children were not significantly different from those of adults, t(102) = 0.579, *p* = 0.564 (see Table 1). There were no differences between children and adults in the motor impulsivity, t(102) = 0.078, *p* = 0.938, attentional impulsivity, t(102) = 0.661, *p* = 0.510, and non-planning impulsivity, t(102) = −0.276, *p* = 0.783, and the whole scale, t(102) = 0.177, *p* = 0.859 (see Table 1).

**Table 1:** Means and standard errors (SEs) of Raven’s scores and the scores of BIS-11 in children and adults.

	Children	Adults	t-value	*p*
Raven	64.29 (3.68)	60.94 (4.53)	0.579	0.564
BIS- motor	24.36 (1.04)	24.25 (0.86)	0.078	0.938
BIS- attentional	23.54 (1.04)	22.69 (0.67)	0.661	0.510
BIS- non-planning	23.36 (1.11)	23.75 (0.83)	− 0.276	0.783
BIS total	71.25 (2.37)	70.69 (2.02)	0.177	0.859

**Figure 1:**
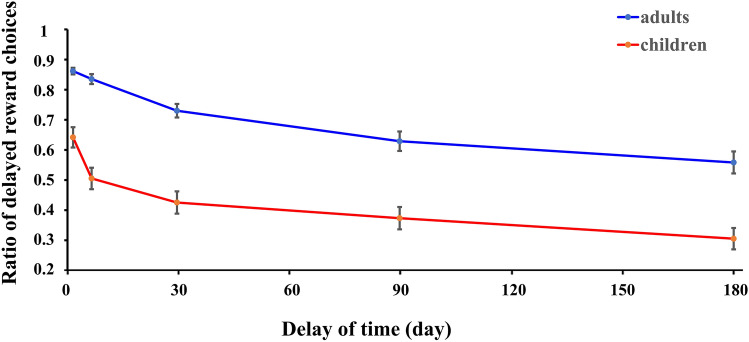
A comparison between children and adults on the ratio of delayed reward choices for various time delays. The picture was created by Microsoft Excel 2016 MSO (16.0.13628.20234) 64 bits (https://www.microsoft.com/zh-cn/microsoft-365/excel) and Microsoft PowerPoint 2016 MSO (16.0.13628.20234) 64 bits (https://www.microsoft.com/zh-cn/microsoft-365/powerpoint).

**Figure 2:**
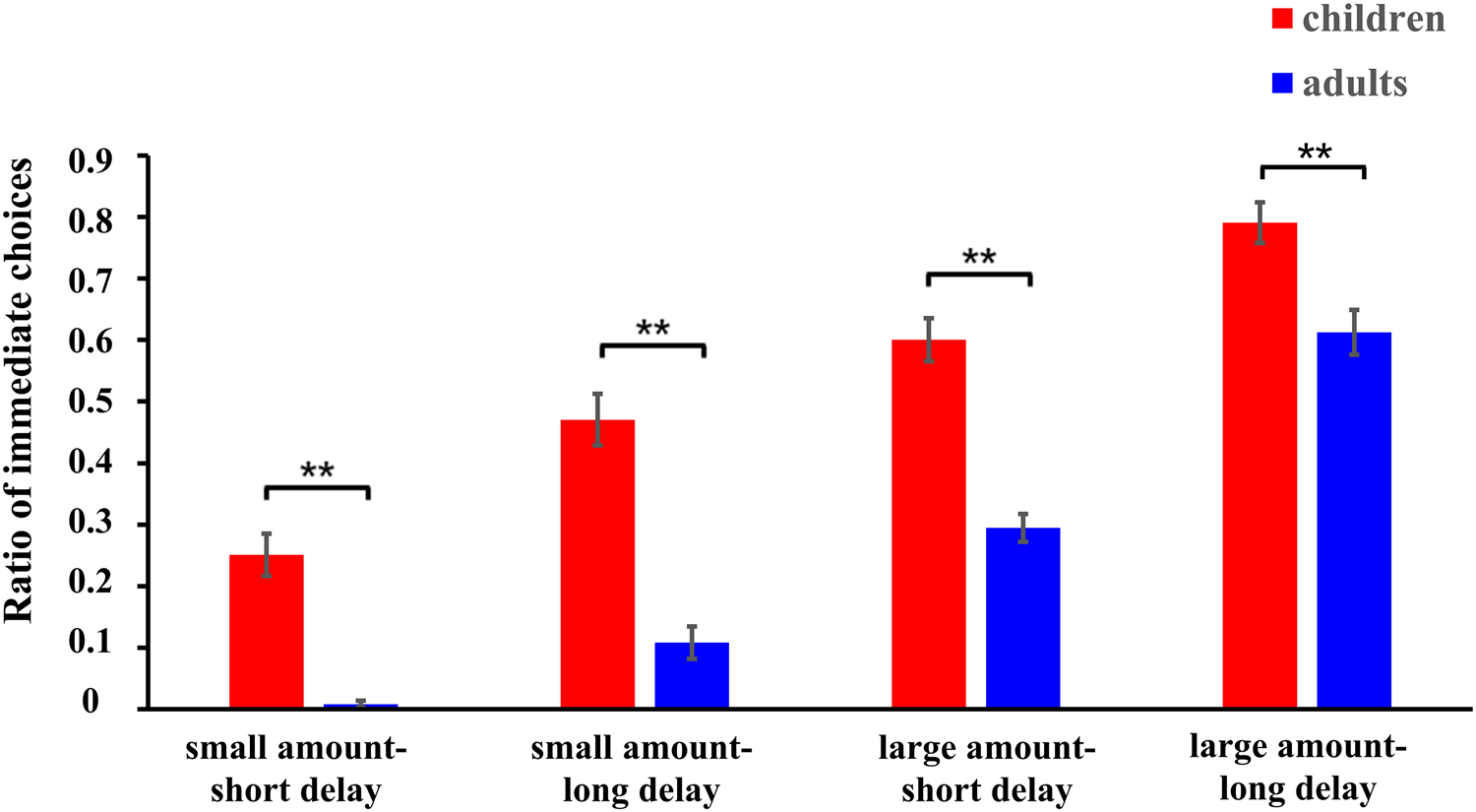
The mean percentage for immediate reward choices during the four conditions in children and adults. The picture was created by Microsoft Excel 2016 MSO (16.0.13628.20234) 64 bits (https://www.microsoft.com/zh-cn/microsoft-365/excel) and Microsoft PowerPoint 2016 MSO (16.0.13628.20234) 64 bits (https://www.microsoft.com/zh-cn/microsoft-365/powerpoint).

To analyze decision-making processes among varied reward amounts and delays in pre-adolescent children and adults (see Fig. 1), the k values, ln(k), RTs and ratios of immediate choices were calculated. The means and standard errors of these variables are presented in Table 2. The different ratios of delayed reward choices of children and adults for varied delays of time are displayed in Fig. 2. The ANOVA results on the RTs and the ratios of immediate choices are shown in Tables 3 and 4.

**Table 2:** Means and standard errors (SEs) of k, ln(k), RT and the ratio of immediate choices in children and adults.

	RT	k(SE)	ln(k)(SE)	Ratio (SE)
**Children**				
Female	2295 (133)	0.28 (0.07)	− 2.35 (0.38)	0.54 (0.05)
Male	1985 (87)	0.33 (0.09)	− 2.40 (0.33)	0.55 (0.04)
Total	2113 (77)	0.31 (0.06)	− 2.38 (0.25)	0.55 (0.03)
**Adult**				
Female	1789 (176)	0.01 (0.003)	− 4.86 (0.19)	0.23 (0.02)
Male	1978 (149)	0.02 (0.01)	− 4.43 (0.24)	0.32 (0.03)
Total	1887 (114)	0.02 (0.004)	− 4.64 (0.16)	0.28 (0.02)

**Table 3:** Main and interaction effects in the ANOVA analyses for reaction time.

	F	p	*ƞ*^2^
Age	3.98	0.049*	0.04
Gender	0.13	0.72	0.001
Age × Gender	3.37	0.07	0.03
Reward amount	9.47	0.003**	0.09
Reward amount × Age	7.90	0.006**	0.07
Reward amount × Gender	0.84	0.36	0.01
Time delay	11.19	0.001**	0.10
Time delay × Age	1.41	0.24	0.01
Time delay × Gender	2.01	0.16	0.02
Reward amount × Time delay	11.18	0.001**	0.10

*Significance ≤ 0.05; **Significance ≤ 0.01.

**Table 4:** Main and interaction effects in the ANOVA analyses for the ratio of immediate choices.

	F	p	*ƞ*^2^
Age	46.57	< 0.001**	0.31
Gender	1.03	0.31	0.01
Age × Gender	0.93	0.34	0.01
Reward amount	660.44	< 0.001**	0.87
Reward amount × Age	4.09	0.046*	0.04
Reward amount × Gender	0.07	0.79	0.001
Time delay	151.10	< 0.001**	0.60
Time delay × Age	0.03	0.86	0.00
Time delay × Gender	4.51	0.036*	0.04
Reward amount × Time delay	25.86	< 0.001**	0.20
Reward amount × Time delay × Age	47.46	< 0.001**	0.32

*Significance ≤ 0.05, **Significance ≤ 0.01.

Regarding repeated measures analysis of RTs, a main effect of age was marginally significant, *F*(1, 102) = 3.98, *p* = 0.049, *ƞ*^2^ = 0.04, in which the RTs of the children were longer than those of the adults. The main effect of reward amount was also significant, *F*(1, 102) = 9.47, *p* = 0.003, *ƞ*^2^ = 0.09, in which the RT of the large amount was longer than that of the small amount. The main effect of time delay was also significant, *F*(1, 102) = 11.19, *p* = 0.001, *ƞ*^2^ = 0.10, in which the RTs of short time delays were longer than those of long time delays. The results showed that children might be slower in choice making and both children and adults in large amount or short time delay spent more time considering choices than in small amount or long time delay.

The interaction effect between age and reward amount was significant, *F*(1, 102) = 7.90, *p* = 0.006, *ƞ*^2^ = 0.07. After the simple-effect tests, it was found that children had longer RTs in the large amount reward condition than in the small amount reward condition (*p* < 0.001), while adults showed similar RTs in both the large and small amount conditions (*p* = 0.86 > 0.05). It was also found that children had significantly longer RTs than adults in the large amount condition (mean_children_ = 2227.12, mean_adults_ = 1890.05, *p* = 0.018) but not in the small amount condition (mean_children_ = 2081.27, mean_adults_ = 1883.48, *p* = 0.14). The results showed that children spent more time in large amount condition, which showed that they cared about large amount more than adults. The interaction effect between reward amount and time delay was also significant, *F*(1, 102) = 11.18, *p* = 0.001, *ƞ*^2^ = 0.10, and it was observed that in the small amount condition, the RTs of a short time delay were longer than those of a long time delay (mean_short delay_ = 2032.95, mean_long delay_ = 1931.79, *p* < 0.001), while in the large amount condition, there was no significant difference between the RTs of short and long time delays (mean_short delay_ = 2067.77, mean_long delay_ = 2049.40, *p* = 0.40). Additionally, for long time delays, the RTs in the large amount condition were longer than those in the small amount condition (*p* < 0.001), while there were no differences between these two conditions for short time delays (*p* = 0.22). The results indicated that participants needed more time in small amount and short delay condition to consider than in small amount and long delay condition. Besides, participants spent more time in large amount and long delay condition to decide than in small amount and long delay condition.

For the univariate ANOVA of ln(k) values, only the main effect of age was significant, *F*(1, 102) = 54.04, *p* < 0.001, *ƞ*^2^ = 0.35, in which the ln(k) values of children were larger than those of adults (mean_children_ = −2.37, mean_adults_ = −4.64). There were no other main or interaction effects on ln(k) values. The results indicated that children discounted more than adults.

For the repeated measures analysis of the ratio of immediate choices, the main effect of age was significant, *F*(1, 102) = 46.57, *p* < 0.001, *ƞ*^2^ = 0.31, in which children chose more immediate choices than adults (mean_children_ = 0.53, mean_adults_ = 0.25). The main effect of reward amount was also significant, *F*(1, 102) = 660.44, *p* < 0.001, *ƞ*^2^ = 0.87, in which the ratio of immediate choices in the large amount condition was larger than that in the small amount condition (mean_small amount_ = 0.21, mean_large amount_ = 0.57). The main effect of time delay was also significant, *F*(1, 102) = 151.10, *p* < 0.001, *ƞ*^2^ = 0.60, in which the ratio of immediate choices in the long time delay condition was larger than that in the short time delay condition (mean_short delay_ = 0.29, mean_long delay_ = 0.49). The results showed that children discounted more than adults and when the immediate reward was large or the delayed time was long, participants would more likely to choose immediate choices.

Furthermore, Spearman correlation analysis showed that the correlation between ln(k) values and the ratio of immediate choice was significant for children (r (58) = 0.98, *p* < 0.001) and adults (r (48) = 0.95, *p* < 0.001). The larger the ln(k) values was, the more immediate choices individuals would make. The significant results showed consistency across the different analytical methods.

### ERP results

**Figure 3:**
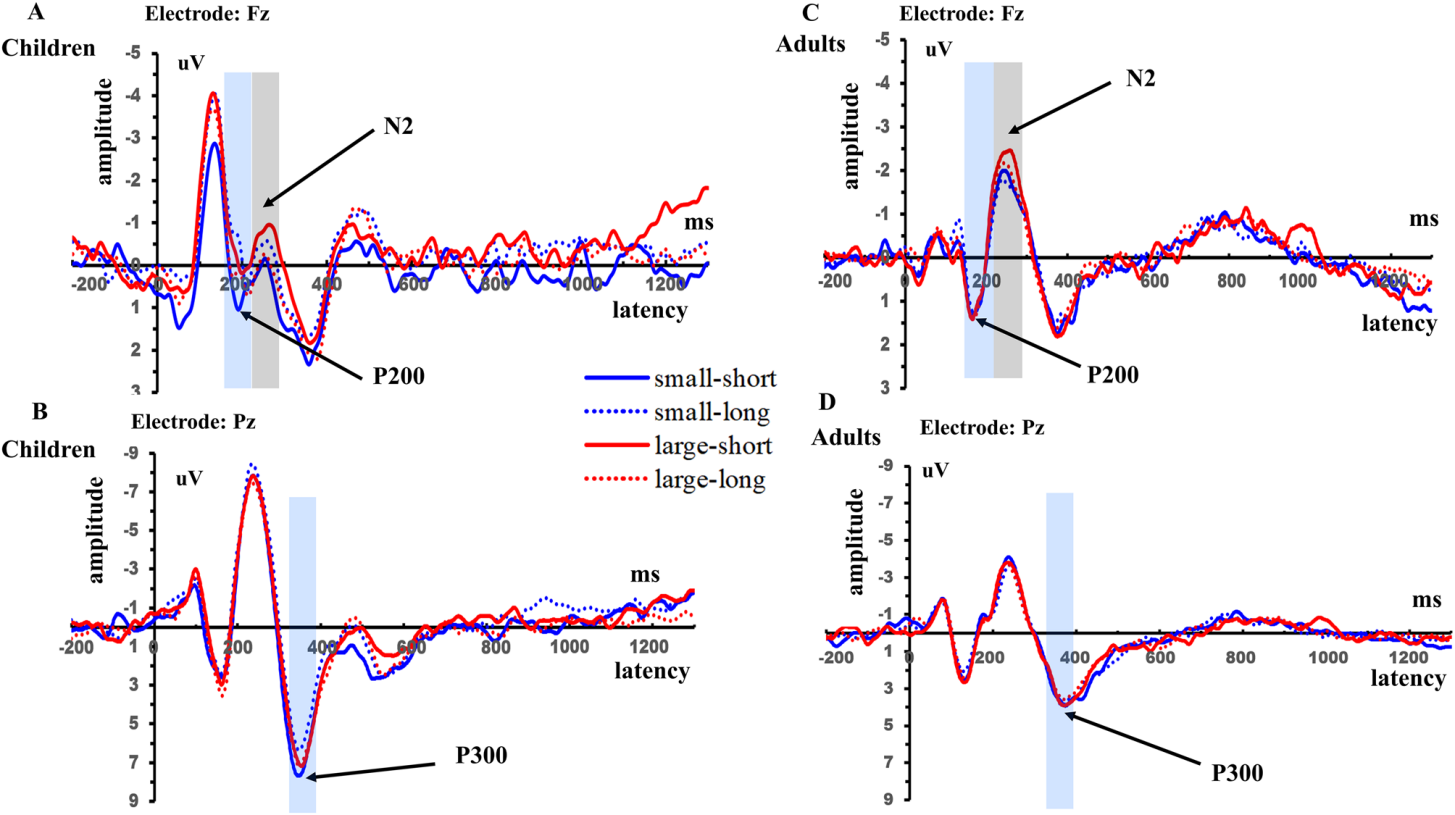
The grand-averaged N2 and P2 ERP waveforms at electrode Fz (**A**) and P3 waveforms at electrode Pz (**B**) in children. (**C**) and (**D**) shows the N2 and P2 waveforms at Fz and P3 waveforms at Pz in adults. The picture was created by Microsoft Excel 2016 MSO (16.0.13628.20234) 64 bits (https://www.microsoft.com/zh-cn/microsoft-365/excel) and Microsoft PowerPoint 2016 MSO (16.0.13628.20234) 64 bits (https://www.microsoft.com/zh-cn/microsoft-365/powerpoint).

**Figure 4:**
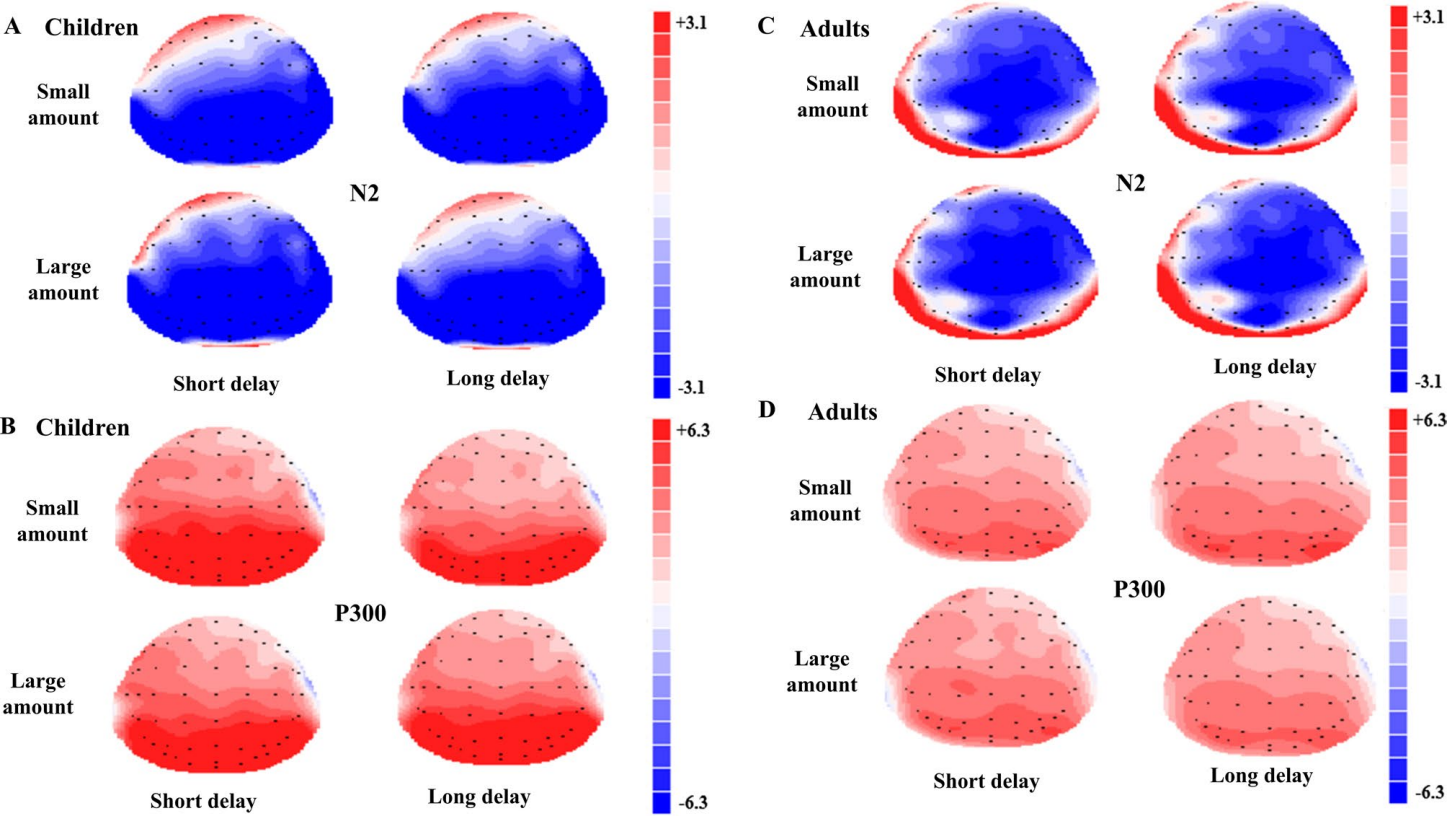
The topographic maps for N2 (**A**) and P3 (**B**) components in children. (**C**) and (**D**) shows the topographic maps for N2 and P3 components in adults. The picture was created by Scan 4.5 (http://www.neuroscan.com/) and Microsoft PowerPoint 2016 MSO (16.0.13628.20234) 64 bits (https://www.microsoft.com/zh-cn/microsoft-365/powerpoint).

The means and standard errors (SEs) of the latencies and amplitudes of P2, N2 and P3 components are shown in Table 5. We presented only the age-related ANOVA results and hypothesis-relevant post-hoc analyses in the text; all ANOVA results for the amplitudes and latencies are shown in Tables 6 and 7. The grand average waveforms of these maximum amplitudes for ERP components are displayed in Fig. 3, and the topographic maps are presented in Fig. 4.

**Table 5:** Means and standard errors of ERP latencies and amplitudes in children and adults.

	P2		N2		P3	
	Latency	Amplitude	Latency	Amplitude	Latency	Amplitude
**Children**						
SS	198.24 (2.98)	0.29 (0.37)	245.28 (3.61)	− 0.22 (0.38)	354.77 (3.32)	5.94 (0.39)
SL	201.69 (2.94)	− 0.57 (0.33)	243.40 (3.50)	− 0.71 (0.35)	357.02 (3.11)	4.72 (0.32)
LS	199.01 (3.00)	− 0.44 (0.32)	249.96 (3.43)	− 0.94 (0.40)	358.69 (3.03)	5.57 (0.39)
LL	199.77 (3.01)	− 0.03 (0.34)	238.16 (3.55)	− 0.38 (0.41)	355.88 (2.81)	5.64 (0.33)
**Adults**						
SS	179.30 (3.23)	0.49 (0.40)	251.27 (3.91)	− 1.55 (0.41)	370.28 (3.60)	3.18 (0.42)
SL	181.63 (3.18)	0.49 (0.35)	251.25 (3.79)	− 1.44 (0.38)	368.78 (3.37)	3.11 (0.34)
LS	174.83 (3.25)	0.29 (0.35)	248.64 (3.71)	− 2.06 (0.44)	368.45 (3.29)	3.19 (0.43)
LL	178.23 (3.26)	0.46 (0.37)	248.13 (3.85)	− 1.70 (0.45)	370.69 (3.04)	3.01 (0.36)

*SS* Small amount and short delay condition, *SL* small amount and long delay condition, *LS* large amount and short delay condition, *LL* large amount and long delay condition.

**Table 6:** Main and interaction effects in ANOVA analyses for ERP amplitudes.

Factors	P2 amplitude			N2 amplitude			P3 amplitude		
	F	p	*ƞ*^2^	F	p	*ƞ*^2^	F	p	*ƞ*^2^
Age	2.27	0.14	0.02	5.15	0.025*	0.05	27.00	< 0.001**	0.21
Gender	1.45	0.23	0.01	0.22	0.64	0.002	3.54	0.06	0.03
Reward amount	0.44	0.51	0.004	2.65	0.11	0.03	0.54	0.46	0.01
Time delay	0.14	0.71	0.001	0.87	0.35	0.01	4.54	0.036*	0.04
Hemisphere	18.36	< 0.001**	0.15	50.88	< 0.001**	0.33	6.32	0.002**	0.06
Age × Gender	0.32	0.57	0.003	0.45	0.50	0.004	0.02	0.88	0.000
Age × Hemisphere	4.37	0.014*	0.04	3.59	0.029*	0.03	4.35	0.014*	0.04
Reward amount × Age	0.00	0.95	0.000	0.29	0.59	0.003	1.00	0.32	0.01
Reward amount × Gender	0.10	0.75	0.001	0.17	0.68	0.002	0.03	0.86	0.000
Reward amount × Time delay	6.33	0.013*	0.06	4.00	0.048*	0.04	3.62	0.06	0.03
Time delay × Age	0.69	0.41	0.01	0.51	0.48	0.01	1.86	0.18	0.02
Time delay × Gender	0.56	0.46	0.01	2.17	0.14	0.02	0.02	0.90	0.000
Age × Reward amount × Gender	5.57	0.020*	0.05	3.21	0.08	0.03	3.49	0.07	0.03
Age × Time delay × Gender	0.003	0.95	0.000	1.79	0.18	0.02	1.12	0.29	0.01
Age × Time delay × Reward amount	3.79	0.054	0.04	1.53	0.22	0.02	5.07	0.027*	0.05

*Significance ≤ 0.05; **Significance ≤ 0.01.

**Table 7:** Main and interaction effects in the ANOVA analyses for ERP latencies.

Factors	P2 latencies			N2 latencies			P3 latencies		
	F	p	*ƞ*^2^	F	p	*ƞ*^2^	F	p	*ƞ*^2^
Age	31.89	< 0.001**	0.24	1.65	0.20	0.02	12.58	0.001**	0.11
Gender	0.21	0.65	0.002	2.26	0.14	0.02	0.26	0.61	0.003
Reward amount	3.61	0.06	0.03	1.19	0.28	0.01	0.21	0.65	0.002
Time delay	3.01	0.09	0.03	4.33	0.04	0.04	0.001	0.97	0.000
Hemisphere	8.80	< 0.001**	0.08	7.57	0.001**	0.07	2.93	0.055	0.03
Age × Gender	0.11	0.74	0.001	1.50	0.22	0.01	0.80	0.37	0.01
Age × Hemisphere	1.87	0.16	0.02	2.35	0.10	0.02	4.44	0.013**	0.04
Reward amount × Age	2.01	0.16	0.02	0.80	0.37	0.01	0.18	0.67	0.002
Reward amount × Gender	0.37	0.54	0.004	1.55	0.22	0.02	0.72	0.40	0.01
Reward amount × Time delay	0.09	0.76	0.001	2.43	0.12	0.02	0.04	0.84	0.000
Time delay × Age	0.07	0.79	0.001	3.72	0.06	0.04	0.05	0.82	0.001
Time delay × Gender	0.32	0.57	0.003	3.93	0.05	0.04	6.36	0.013*	0.06
Age × Reward amount × Gender	1.26	0.26	0.01	0.93	0.34	0.01	3.30	0.07	0.03
Age × Time delay × Gender	0.14	0.71	0.001	0.59	0.45	0.01	10.90	0.001**	0.10
Age × Time delay × Reward amount	0.49	0.485	0.01	2.00	0.16	0.02	1.83	0.18	0.02

*Significance ≤ 0.05; **Significance ≤ 0.001.

### Age effects in neural activity

Significant age effect was found in P2 latencies, N2 amplitudes, P3 amplitudes and latencies. For P2 latencies, the main effect of age was significant, *F*(1, 102) = 31.89, *p* < 0.001, *ƞ*^2^ = 0.24, in which children had longer P2 latencies than adults (mean_children_ = 199.68, mean_adults_ = 178.50). With regard to N2 amplitudes, the main effect of age was significant, *F*(1, 102) = 5.15, *p* = 0.025, *ƞ*^2^ = 0.05, in which the amplitudes of children were smaller than those of adults (mean_children_ = − 0.56, mean_adults_ = − 1.69). About P3 amplitudes, the main effect of age was significant, *F*(1, 102) = 27.00, *p* < 0.001, *ƞ*^2^ = 0.21, in which children had larger P3 amplitudes than adults (mean_children_ = 5.45, mean_adults_ = 3.12). For P3 latencies, the main effect of age was significant, *F*(1, 102) = 12.58, *p* = 0.001, *ƞ*^2^ = 0.11, in which children had shorter P3 latencies than adults (mean_children_ = 356.59, mean_adults_ = 369.55,* p* = 0.001). The results showed that children exhibited longer P2 latencies, smaller N2 amplitudes and larger and shorter P3 amplitudes than adults.

### Reward amount and time delay effects on age in neural activity

For P2 amplitudes, the interaction effect of reward amount, time delay and age was marginally significant, *F*(1, 102) = 3.79, *p* = 0.054, *ƞ*^2^ = 0.04. In the small amount and long delay condition, P2 amplitudes of children were smaller than those of adults (mean_children_ = − 0.57, mean_adults_ = 0.49, *p* = 0.046), while in the small amount and short delay condition, in large amount and short delay condition and in large amount and long delay condition, P2 amplitudes of children were not different from those of adults (*ps* > 0.05). The interaction effect of age, gender and reward amount was significant, *F*(1, 102) = 5.57, *p* = 0.02, *ƞ*^2^ = 0.05. However, after a simple-effect test, no significant comparisons were found. There was no significant interaction effect of reward amount and/or time delay and age on P2 latencies. The larger P2 amplitudes of adults compared with those of children in small amount and long delay condition showed the age difference in early attention stage.

For N2 amplitudes, the interaction of reward amount, age and gender was marginally significant, *F*(1, 102) = 3.21, *p* = 0.08, *ƞ*^2^ = 0.03. It was further found that in the small amount conditions for males, the N2 amplitudes of children were smaller than those of adults (mean_children_ = − 0.22, mean_adults_ = − 1.90, *p* = 0.012), while no differences were found between the amplitudes of children and adults in the small amount conditions for females or in the large amount conditions for males and females (small amount, female: mean_children_ = −0.71, mean_adults_ = −1.09, *p* = 0.59; large amount, female: mean_children_ = −0.51, mean_adults_ = −1.72, *p* = 0.15; large amount, male: mean_children_ = −0.80, mean_adults_ = −2.04, *p* = 0.10).

For N2 latencies, the interaction effect of age and time delay was marginally significant, *F*(1, 102) = 3.72, *p* = 0.06, *ƞ*^2^ = 0.04. It was further found that for children the latencies of long delay were longer than those of short delay (mean_short delay_ = 247.62, mean_long delay_ = 240.78; *p* = 0.004; *ƞ*^2^ = 0.079). However, for adults, the latencies of short delay were not significantly different from those of long delay (mean_short delay_ = 249.95, mean_long delay_ = 249.69; *p* = 0.917). The results of N2 response indicated that age effects on N2 amplitudes were mainly affected by reward amount while age effects on N2 latencies were mainly influenced by time delay.

For P3 amplitudes, the interaction effect of reward amount, time delay and age was significant, *F*(1, 102) = 5.07, *p* = 0.027, *ƞ*^2^ = 0.05. After the simple effect test, in small amount condition, children had larger P3 amplitudes for the short delay conditions than for the long delay conditions (children, small amount: mean_short_ = 5.94, mean_long_ = 4.72, *p* < 0.001), while in the large amount conditions for children and in small and large amount conditions for adults, P3 amplitudes in the short delay conditions were not different from those in the long delay conditions (children, large amount: mean_short_ = 5.57, mean_long_ = 5.64, *p* = 0.84; adults, small amount: mean_short_ = 3.18, mean_long_ = 3.11, *p* = 0.82; adults, large amount: mean_short_ = 3.19, mean_long_ = 3.01, *p* = 0.59). In addition, for children in the long delay conditions, the amplitudes of small amounts were smaller than those of large amounts (*p* = 0.002), while for children in the short delay conditions and for adults in the short and long delay conditions, there were no significant differences between the amplitudes of small amounts and large amounts (children, short delay, small amount and large amount: *p* = 0.25; adults, short delay, small amount and large amount: *p* = 0.97; adults, long delay, small amount and large amount: *p* = 0.76). In short word, the P3 amplitudes results showed that children exhibited larger amplitudes in small amount and short delay condition and in large amount and long delay condition compared with small amount and long delay condition while adults showed comparable amplitudes in these conditions.

For P3 latencies, the interaction effect of reward amount, age and gender was marginally significant, *F*(1, 102) = 3.30, *p* = 0.07, *ƞ*^2^ = 0.03. After a simple effect test, it was found that all comparison pairs except in large amount condition for females were significant (small amount: mean_female children_ = 357.69, mean_female adults_ = 370.92, *p* = 0.043; mean_male children_ = 354.09, mean_male adults_ = 368.14, *p* = 0.018; large amount: mean_female children_ = 360.61, mean_female adults_ = 366.76, *p* = 0.249; mean_male children_ = 353.95, mean_male adults_ = 372.380, *p* < 0.001). The results indicated there were no significant age differences on P3 latencies in large amount condition for females while in other conditions there were significant age differences.

For P3 latencies, the interaction effect of time delay, gender and age was also significant, *F*(1, 102) = 10.90, *p* = 0.001, *ƞ*^2^ = 0.10. A simple effect test revealed that in short delay condition, the P3 latencies of female children were shorter than those of female adults, the latencies of male children were shorter than those of male adults and in long delay condition, the P3 latencies of male children were shorter than those of male adults (short delay: mean_female children_ = 358.73, mean_female adults_ = 372.82, *p* = 0.018; mean_male children_ = 354.72, mean_male adults_ = 365.90, *p* = 0.037; long delay: mean_female children_ = 359.57, mean_female adults_ = 364.86, *p* = 0.364; mean_male children_ = 353.33, mean_male adults_ = 374.61, *p* < 0.001). The results indicated there were no significant age differences on P3 latencies in long delay condition for females while in other conditions there were significant age differences.

### Hemisphere effects in neural activity

We mainly consider the interaction effect of age and hemisphere according to our hypothesis. The significant interaction effects of hemisphere and age were in the amplitudes of P2, N2 and P3 and in the latencies of P3.

With regard to P2 amplitudes, the interaction effect of age and hemisphere was significant, *F*(2, 204) = 4.37, *p* = 0.014 , *ƞ*^2^ = 0.04. After the simple-effect test, the amplitudes of children on the right side were significantly smaller than those of adults (children: mean_left_ = −0.05, mean_right_ = −0.75, mean_middle_ = 0.24; adults: mean _left_ = 0.69, mean_right_ = 0.15, mean_middle_ = 0.46), while the amplitudes of children were statistically comparable to those of adults in the left and middle brain areas (*p*(left) = 0.086; *p* (middle) = 0.626; *p* (right) = 0.038, *ƞ*^2^ = 0.042). The response of P2 component showed that the immaturity of hemisphere on age in this neural stage related to early detection was mainly in the right side.

With regard to N2 amplitudes, the interaction effect of age and hemisphere was significant, *F*(2, 204) = 3.59, *p* = 0.029, *ƞ*^2^ = 0.03. After a simple-effect test, the N2 amplitudes of children were significantly smaller than adults over the middle and right areas (children: mean_left_ = −0.01, mean_right_ = −1.01, mean_middle_ = −0.66; adults: mean_left_ = −0.77, mean_right_ = −2.20, mean_middle_ = −2.09), while the activities of both age groups were not significantly different on the left brain areas (*p* (right) = 0.017; *p* (middle) = 0.008; *p* (left) = 0.16). The response of N2 indicated that the lag by age was on the right in the stage related to cognitive control.

With regard to the P3 amplitudes, the interaction effect between age and hemisphere was significant, F(2, 204) = 4.35, *p* = 0.014, *ƞ*^2^ = 0.04. After a simple-effect test, it was found that for children the amplitudes in the middle part were significantly smaller than those in the left side or right side while the amplitudes of the left and right were comparable (mean_left_ = 5.57, mean_right_ = 5.86, mean_middle_ = 4.97, *p*(left and right) = 0.644, *p*(left and middle) = 0.016, *p*(right and middle) < 0.001). For adults, the amplitudes of the three parts were not significantly different from each other (adults: mean_left_ = 3.33, mean_right_ = 3.03, mean_middle_ = 3.01; *p*(left and right) = 0.72, *p*(left and middle) = 0.51, *p*(right and middle) = 1.000). Meanwhile, the amplitudes of left, right and middle parts of children were all significantly larger than those of adults (*p* (left) < 0.001, *ƞ*^2^ = 0.19; *p* (right) < 0.001, *ƞ*^2^ = 0.28; *p*(middle) < 0.001, *ƞ*^2^ = 0.12).

With regard to P3 latencies, the interaction effect of age and hemisphere was also significant, *F*(2, 204) = 4.44, *p* = 0.013, *ƞ*^2^ = 0.04. After a simple-effect test, it was observed that for children, the latencies of the left side were longer than those of the middle and right sides while there were no difference between the latencies of the right and middle sides (mean_left_ = 359.90, mean_right_ = 353.83, mean_middle_ = 356.04; *p*(left and right) = 0.002, *p*(left and middle) = 0.051, *p*(right and middle) = 0.40). For adults, the latencies of the left, middle and right sides were comparable (mean_left_ = 368.85, mean_right_ = 369.27, mean_middle_ = 370.52; *p*(left and right) = 1.00, *p*(left and middle) = 1.00, *p*(right and middle) = 1.00). Meanwhile, the latencies in the left, middle and right sides were all longer in adults than those in children (*p* (left) = 0.032, *ƞ*^2^ = 0.045; *p* (right) < 0.001, *ƞ*^2^ = 0.14; *p*(middle) < 0.001, *ƞ*^2^ = 0.13). The results showed that children exhibited larger and shorter P3 in the three brain parts in the information processing stage.

### Correlations between behavioral and ERP results

**Figure 5:**
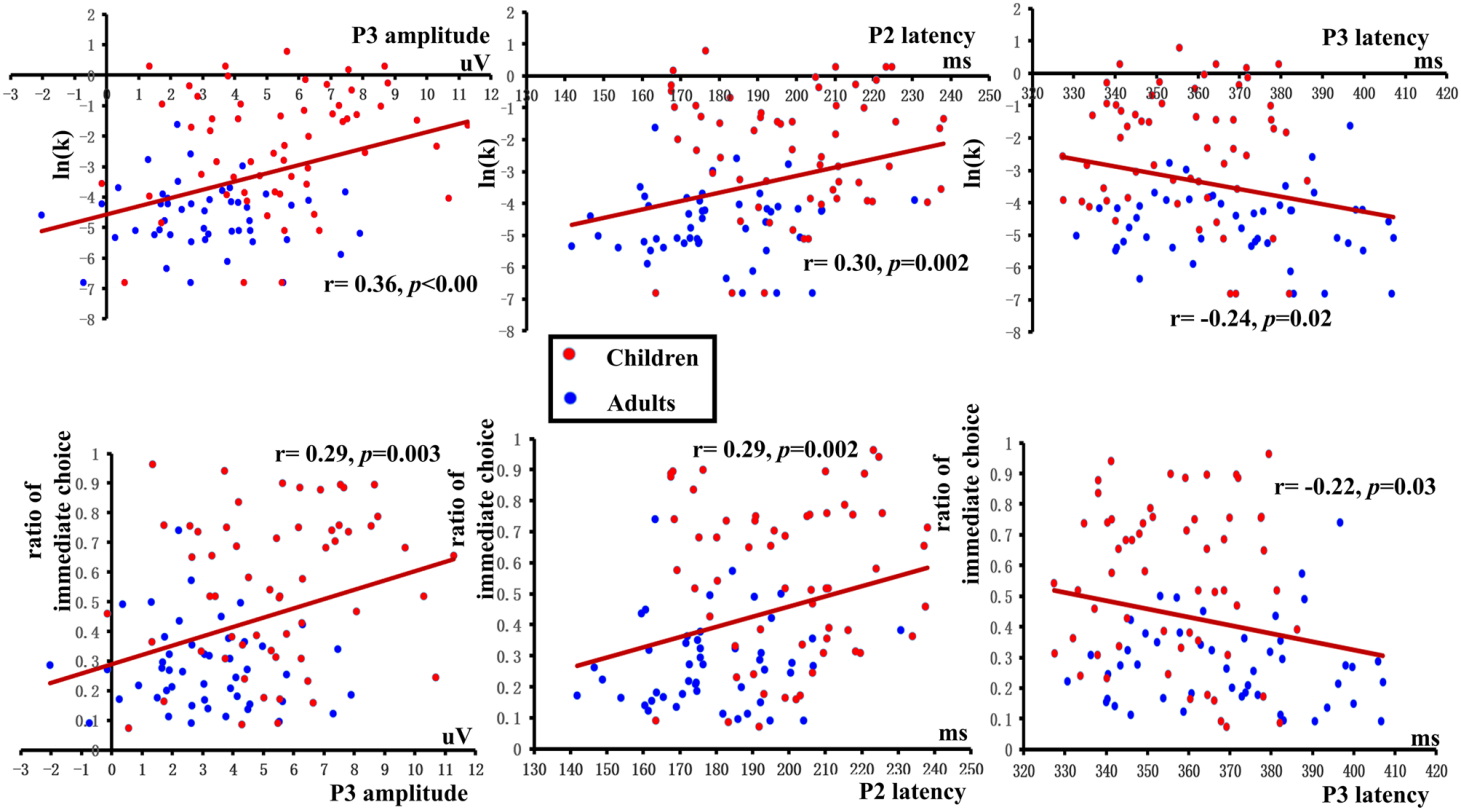
The correlations of ln(k)(the top row)/ ratio (the bottom row) and P2 latencies, P3 amplitudes and latencies in the whole group. The picture was created by Microsoft Excel 2016 MSO (16.0.13628.20234) 64 bits (https://www.microsoft.com/zh-cn/microsoft-365/excel) and Microsoft PowerPoint 2016 MSO (16.0.13628.20234) 64 bits (https://www.microsoft.com/zh-cn/microsoft-365/powerpoint).

The correlations between the ratio of immediate choices and ERP responses and that between the ln(k) values and ERP responses are presented in Table 8. There were significant correlations between P3 amplitudes and ln(k) values (*r* (106) = 0.36, *p* < 0.001), between P3 amplitudes and the ratio of immediate choices (*r* (106) = 0.29, *p* = 0.003) and between P3 latencies and ln(k) values (*r* (106) = −0.24, *p* = 0.02), and between P3 latencies and the ratio of immediate choices (*r* (106) = −0.22, *p* = 0.03). Moreover, there were significant differences between P2 latencies and ln(k) values (*r* (106) = 0.30, *p* = 0.002) and between P2 latencies and the ratio of immediate choices (*r* (106) = 0.29, *p* = 0.002). The scatter-plots of the significant correlations are presented in Fig. 5. From the scatter-plots, we can find that the mean values of children’s ratio or ln(k) were different from those of adults’, as well as the amplitudes or latencies of ERP components. This showed that the significant correlations of behavior results and ERP components were spurious. The results showed that there were no real significant correlations of behavioral results and ERP potentials.

**Table 8:** Pearson’s correlations among ln(k) values, ERP component amplitudes and ERP component latencies, and Spearman’s correlations among the ratio of immediate choices, ERP component amplitudes and ERP component latencies.

	Children (n = 58)		Adults (n = 48)		Total sample (n = 106)	
	ln(k)	Ratio	ln(k)	Ratio	ln(k)	Ratio
N2 amplitude	− 0.07 (0.61)	− 0.07 (0.58)	0.22 (0.14)	0.24 (0.09)	0.15 (0.13)	0.12 (0.24)
P2 amplitude	0.00 (0.998)	− 0.06 (0.68)	− 0.06 (0.69)	− 0.01 (0.96)	− 0.10 (0.31)	− 0.15 (0.12)
P3 amplitude	0.21 (0.11)	0.20 (0.14)	− 0.05 (0.73)	− 0.09 (0.54)	0.36 (<0.001)**	0.29 (0.003)**
N2 latency	− 0.05 (0.69)	− 0.12 (0.39)	− 0.18 (0.22)	− 0.15 (0.32)	− 0.15 (0.14)	− 0.19 (0.05)
P2 latency	0.009 (0.95)	0.01 (0.94)	0.03 (0.84)	0.13 (0.39)	**0.30 **(0.002)**	**0.29 **(0.002)**
P3 latency	− 0.05 (0.70)	− 0.02 (0.88)	− 0.05 (0.76)	− 0.08 (0.61)	**− 0.24 **(0.02)*	**− 0.22 **(0.03)*

*Significance ≤ 0.05; **Significance ≤ 0.01.

## Methods

### Participants

The study was approved by the local Ethics Committee of the Institute of Psychology, Chinese Academy of Science and was conducted in accordance with the Declaration of Helsinki. Two age groups participated in the current study. Sixty-two children were recruited from a local elementary school by advertisement. Two children were excluded due to recording errors of the instrument, one child was excluded due to attention problems, and one child was excluded because there were not enough trials suitable for neural analysis. Thus, 58 children (34 boys; 9–10 years old; mean age: 9.26 ± 0.44 years) were included for further analysis. For the adult group, 50 adults were recruited from local universities by advertisement; one adult was excluded due to a different sampling rate, and one adult was excluded due to a history of depression. Therefore, there were 48 adults (25 males; aged 18–26 years old; mean age: 20.98 ± 1.93 years) included for further analysis. Child participants were awarded for participation with a present after completion of the task, and adult participants were paid for participation. All participants were right-handed with normal or corrected-to-normal vision, reported being free from psychiatric problems, and did not regularly use medications. Written informed consent was provided by parents of the children and by adult participants before the experiment.

We asked the adult participants to rate their desirability of obtaining the money rewards using a seven-point scale with larger numbers showing a larger degree of desire before and after the experiment. The degree of desirability of all adults were equal or greater than 4 (mean_before_ = 6.10 ± 0.71, mean_after_ = 6.29 ± 0.64).

### Materials

The Raven’s Standard Progressive Matrices (RSPM)^57^ were used to assess participants’ mental ability associated with abstract reasoning and has little dependency on language abilities. It consists of 60 items and has been used as an indicator of general intelligence throughout the world^59^. Chinese psychologists revised the scale’s norms and proved the scale to have good reliability and validity in Chinese sample^60^.

We used a Chinese revised version of Barratt Impulsiveness Scale, Version 11^58^ to measure participants’ trait impulsivity. The questionnaire has been verified to have good construct convergent, and discriminant validity^61^. It is consisted of 30 items and has three subscales: motor impulsivity, attentional impulsivity and non-planning impulsivity. It has been adapted to a five-point scale, and the cognitive and unplanned impulsivity subscales are scored in reverse. High scores indicate hyperactivity, inattention and lack of planning.

### Experimental stimuli and paradigm

The current task was an adaption of Mitchell’s paradigm^12^. The participants were seated comfortably in front of a computer and performed the task in an electromagnetically shielded room. The stimuli were presented on a computer monitor with a 60 Hz refresh rate, and the acquisition of behavioral data was conducted with E-Prime software (Version 2.0, Psychology Software Tools, Inc.).

For each trial, two options were presented on the right and left sides of the screen simultaneously. One was an immediate and smaller monetary reward, and the other was a delayed and larger monetary reward. Participants were asked to choose one of the two options. The immediate options referred to a situation in which participants could gain the monetary reward immediately after the experiment. The amount of immediate monetary rewards changed from ¥0 to ¥63 in the step of ¥3, in which ¥0 ~ ¥30 were regarded as the small amount and ¥33 ~ ¥63 were regarded as the large amount. The delayed options referred to the situation in which participants could gain a fixed amount of money (¥60 = $9.26) in varied delayed times from 2 to 180 days (detailed delays: 2, 7, 30, 90 or 180 days), in which 2 and 7 days were deemed short time delays and 30, 90 and 180 days were deemed long time delays. The reward amount chosen here was similar to the amount in Mitchell’s study^12^, which was proper for the potential real paradigm and in de Water’s study, which was suitable for the exploration of age differences in delay discounting^62^. The immediate rewards and delayed times were selected randomly to form the choices. Before the formal experiment, there were 8 practice trials, which were used to make subjects understand the rules of the task. In the formal task, there were 44 trials for the small amount (immediate option) and short delay (delay option) condition, 66 trials for the small amount (immediate option) and long delay (delay option) condition, 44 trials for the large amount (immediate option) and short delay (delay option) condition, and 66 trials for the large amount (immediate option) and long delay (delay option) condition. Participants were required to have a 2–3 min rest period every 74 trials.

**Figure 6:**
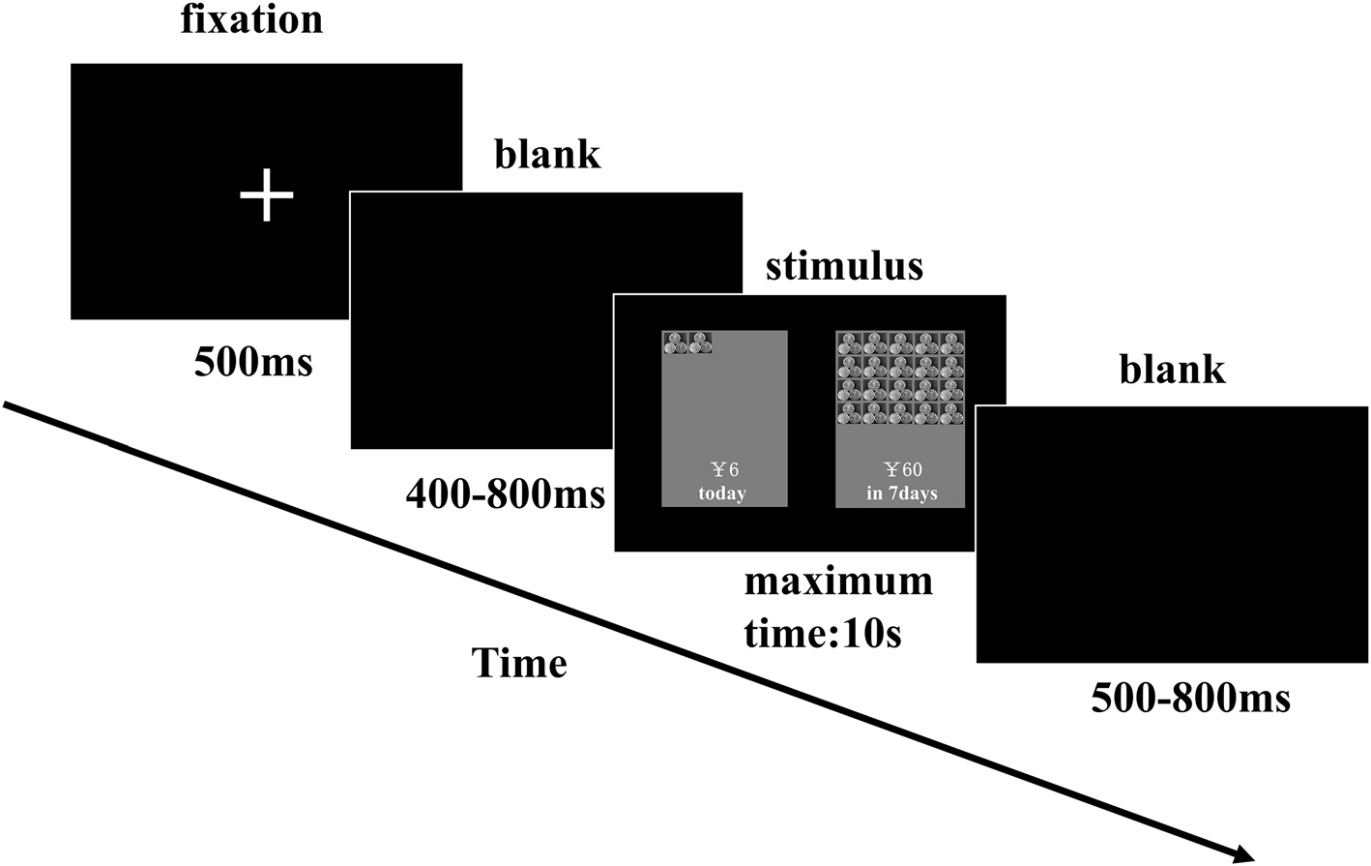
The procedure of the delay discounting tasks. The picture was created by Microsoft PowerPoint 2016 MSO (16.0.13628.20234) 64 bits (https://www.microsoft.com/zh-cn/microsoft-365/powerpoint).

The procedure is presented in Fig. 6. Each trial began with the presentation of a fixation point for 500 ms, followed by a blank screen for a randomized duration ranging from 400 to 800 ms. Then, the choice screen was presented with a maximum time of 10 s, which was long enough for all child and adult participants to make a choice. The intertrial interval was a random period of 500–800 ms. The participants indicated their choices by pressing 'z' with their left index fingers if they preferred the option on the left side of the screen and '/' with their right index fingers if they preferred the option on the right side of the screen. The presentation of the choice pairs and the location of the immediate and delayed choices in each pair were random.

Before the experiment, we informed all the participants about the reward rules. The rewards in the current task were potentially real. Participants were explicitly required to choose the reward options according to their real preference because they would be given one of their choices randomly after the experiment^11^. Participants clearly knew that they could receive the monetary reward immediately after finishing the experiment if they chose the immediate option. Participants were instructed that if they chose a delayed reward option, the money would be transferred to their bank account (adult participants) or given to them in an envelope by their parents (child participants) on the exact day that they chose in the selection phase.

### ERP data acquisition

Electroencephalograms (EEGs) were recorded from a Neuroscan Quick-Cap (64 scalp sites) according to the International 10/20 system, with an online reference to the nose. Horizontal electrooculogram (HEOGs) were recorded from electrodes placed approximately 1.5 cm lateral to the left and right external canthi. Vertical electrooculograms (VEOGs) were recorded from the left supraorbital and infraorbital electrodes. All interelectrode impedances were maintained below 10 kΩ. All signals were sampled at 1000 Hz and online-bandpass filtered within a 0.05–100 Hz frequency range.

For off-line analysis, EEG signals affected by body movement were removed from further analyses, and ocular artifacts were corrected with an eye-movement correction algorithm implemented in Neuroscan software^63^. Trials containing EEG sweeps with amplitudes exceeding ± 90 µV were excluded. EEG data were filtered from 1 to 30 Hz (24 dB/oct). EEG signals were divided into epochs of 1500 ms (together with 200 ms prestimulus as the baseline), time locked to the onset of stimulus. The trial numbers left for further statistical analysis in each condition were as follows. For children, there were 35 ± 6 trials in the small amount and short delay condition, 52 ± 9 trials in the small amount and long delay condition, 34 ± 6 trials in the large amount and short delay condition and 52 ± 8 trials in the large amount and long delay condition. For adults, there were 42 ± 3 trials in the small amount and short delay condition, 63 ± 4 trials in the small amount and long delay condition, 41 ± 3 trials in the large amount and short delay condition, and 63 ± 4 trials in the large amount and long delay condition.

### Data analysis

For the Raven’s Standard Progressive Matrices (RSPM), we converted the original scores to normal scores first and then used independent-samples t test to compare scores of the two age groups. For the Barratt Impulsiveness Scale, Version 11, we calculated the total scores of each subscale and the whole scale and compared them directly between the children and adults by the independent-samples t test. As we found there were no significant differences in intelligence and impulsivity between two age groups, the following analysis did not control them as covariates.

For behavioral data, three parameters were analyzed: the ratio of immediate choices, the discount rate k^13,64^, and the reaction times (RTs) of the choices. The k value was calculated from the hyperbolic model^13^(see Appendix in the supplementary file). We obtained the k value from the turning point, which was the change from delayed choices to immediate choices noted after arranging the immediate qualities of each delay time. V shows the subjective value of an outcome, and A is the objective value of V. D represents the delay to its receipt. In our study, A was fixed at ¥60. D was 2, 7, 30, 90 or 180 days. After entering V, A and D in the formula, we obtained a value for k, the rate of discounting. Because the data of k were not normally distributed and therefore not suitable for further analysis, we transformed k to ln(k), as in a previous study^17^. Notably, ln function is an increasing function in which larger ln values indicate a higher discounting rate and smaller values demonstrate a lower discounting rate. For ln(k) values, univariate ANOVAs were conducted with age (child vs. adult) and gender (female vs. male) as between-subject factors. For RTs and the ratio of immediate choices, repeated measures analyses were conducted separately with reward amount (small amount vs. large amount) and time delay (short delay vs. long delay) as within-subject factors and age (child vs. adult) and gender (female vs. male) as between-subject factors.

For ERP data, three ERP components were analyzed-P2, N2 and P3 components, and the time windows and the electrode sites for these components were chosen based on previous literature^35,65,66^ and visual inspection of the current ERP grand average waveforms. P2 and N2 components were analyzed over the fronto-central areas (Fz, F3, F4, FCz, FC3, and FC4) during the time windows of 140–240 and 200–300 ms, respectively. The P3 component was measured over the centro-parietal electrodes (Pz, P3, P4, CPz, CP3, and CP4) with a time window of 310–410 ms.

For the amplitudes and latencies of P2, N2 and P3, repeated measures analyses were conducted separately with reward amount (small amount vs. large amount), time delay (short delay vs. long delay) and hemisphere (left vs. middle vs. right) as within-subject factors and age (child vs. adult) and gender (female vs. male) as between-subject factors.

For correlations of behavioral results and ERP data, we performed Pearson’s correlation between ln(k) values and the amplitudes or latencies of the ERP components. Because the ratio of immediate choices was not normally distributed, we performed Spearman’s correlation analyses (nonparametric test) between ln(k) values and the ratio of immediate choices and between the amplitudes or latencies of the ERP components and the ratio of immediate choices.

For all statistical analyses, we used SPSS version 22.0. Significant interactions were analyzed by post hoc simple effects. Partial eta-squared is represented to demonstrate the effect size of the results.

## Discussion

The goal of the study was to discover the temporal dynamics underlying the behavioral differences in delay discounting processes of children and adults. The current study showed that children discounted more than adults in behavioral performance and exhibited longer frontal P2, smaller N2 and larger and shorter P3 amplitudes than adults. Besides, hemisphere effect of age was mainly on the right side. In addition, we found that reward amount and time delay influenced children and adults differently mainly in the small amount and long delay condition.

### Age-related differences in delay discounting

The behavioral findings showed that children’s discount rate was higher than that of adults and that children made more immediate choices than adults, which was consistent with previous findings^2,22,67^, while their differences in reaction time were marginally significant. Valuation and cognitive control are very important for delay discounting^68^, and these findings might be because children’s two decision making systems develop discrepantly. Compared to adults, children aged 7–11 years old showed higher sensitivity to rewards in a reward valuation task^69^, and they had worse cognitive control function than adults, including working memory and response inhibition^70,71^. Moreover, children may lack experience with long delays and may view the same range of time as longer than adults perceive it to be^22,67^, which may affect their future orientation. Steinberg et al.^2^ found that younger individuals aged 10–16 who showed a higher discount rate exhibited a weaker orientation to the future, especially in the dimensions of temporal orientation and the anticipation of future consequences but not in the dimension of planning ahead. In fact, children’s concept of the future is formed during development^72^. In addition, different aspects of future orientation might have different developing trajectories since different dimensions of future orientation have different neural bases^73^, which may lead to different influences on delay discounting. Regardless, it is likely that children experience long-time delays less than adults, which makes children less future oriented and more likely to choose immediate choices. Overall, children’s steeper discounting may result mainly from aspects of their cognitive ability and experience, but how the two aspects interact needs to be further investigated in the future.

It is believed that those who discount the value of delayed rewards more deeply experience time to have a higher cost and often overestimate the duration of time intervals^74^. A previous study using time reproduction showed that children often overvalue time^75^; in other words, children often overestimate the duration of time. In addition, it was discovered that adults’ time sensitivity was higher than children^76^; that is, adults made fewer errors and estimated time more correctly than children. Therefore, it seems that children’s lower sensitivity to time delays may lead to higher degrees of discounting. According to this hypothesis, when the time delay increases, children may show a higher degree of overestimation when estimating longer delays than when estimating shorter delays, which may lead to a higher degree of discounting in longer delays than in shorter delays. However, our results contradict this hypothesis, as children exhibited comparable differences to adults in both short and long delays. The reason that children’s long time estimation for 2 or 7 days and for 30, 90 or 180 days in our study was comparable may be because children have less experience in long time delays, and the estimation pattern in long time delays is different from that in short time delays, which are often used in the laboratory.

The difference between children and adults in fluid intelligence was not significant, which is consistent with Olson’s study^67^. In Olson’s study^67^, they compared the fluid intelligence of young participant group (children and adolescents) aged between 9 and 17 years old and adult group aged between 18 and 23 years old, and found no differences between the two age groups in the four subtests (Block Design, Vocabulary, Matrix Reasoning and Similarities) of the Wechsler Abbreviated Scale of Intelligence (WASI). However, other researchers found small but significant age differences in participants aged 10–30 years old in the two subtests of WASI^2^. In Olson’s study, they used four subtests of WASI while in Steinberg’s study two of the four subtests were used to represent participants’ fluid intelligence, which might cause different results. Maybe age differences in intelligence occur in some aspects of the intelligence but not in the general intelligence. Besides, in Olson’s study, the age range in an age group was large (from 9 to 17 for the young group and from 18 to 23 for the adult group) while Steinberg classified 7 age groups between 10 and 30 years old and the age range in a group was small. However, specific age groups were not reported for differences in fluid intelligence in Steinberg’s research. More researches should be done to find out whether there were age differences in different aspects of intelligence in age groups with small age range. Furthermore, we found no group difference in impulsivity. This result is in accordance with Steinberg’s study to some degree^2^, which found that age differences were not mediated by differences in impulsivity measured by BIS-11 in a group of participants aged between 10 and 30 years old. We believe that impulsivity might not influence the age differences in delay discounting in our sample.

Neuroimaging studies have found that the activation of the limbic system associated with the midbrain dopamine system, which is part of the “bottom-up” system, is involved in immediately available rewards, while greater relative frontoparietal activation is related to longer-term options^24^, which is the main part of the “top-down” system. In children, the “bottom-up” brain regions related to reward valuation, such as the ventral striatum, develop faster than the “top-down” brain cortexes involved in cognitive control, such as the DLPFC and parietal cortex^11,49,77–79^.

The P2 component is related to early feature encoding on stimuli and attention filtering in the early decision-making period^35,46,80^, which reflects “bottom-up” process in decision making. The present study observed that children had longer P2 responses than adults. A previous study found that high procrastinators exhibited longer P2 responses in delay discounting, which showed abnormal reward encoding^38^. This finding indicated that children might be slower during early encoding in delay discounting.

During delay discounting, the brain areas involved in cognitive control reflecting the “top-down” system are cortexes such as the lateral prefrontal cortex^24,68^. The frontal N2 component is regarded as a neural marker for individual differences in executive functions such as cognitive control^41^. Individuals suffering from earthquakes have been shown to have smaller N2 responses and to have a higher degree of discounting^46^; also, their N2 amplitude was negatively correlated with the ratio of immediate choices^19^. Our current study found that children had smaller N2 amplitudes than adults, which might indicate children’s immature development in cognitive control and is consistent with the slower development of the top-down processing system in decision making^49,77,78^. This finding was also in agreement with the fMRI finding that children’s ability to overcome temptation increased with improvement in functional coupling between the VMPFC, which is related to reward valuation, and brain regions such as the DLPFC, which are involved in behavioral control^81^.

The P3 component, another component related to the “top-down” system, is regarded as a component to investigate various cognitive processes, processing capacity and mental workload^45,66^. In the present study, children had larger P3 responses than adults. This result could be explained as follows. First, in line with several developmental studies, the findings of the current study suggest that children recruited more neural resources to execute decision-making processes than adults^53^. Casey et al.^82^ observed that children exhibited stronger activation in dorsolateral prefrontal areas than adults when accomplishing a go/nogo task. Moreover, it was discovered that during childhood, working memory is still developing^70^ and that delay discounting is negatively correlated with working memory^55^. Thus, it is reasonable that children might need more neural resources than adults in decision making. Second, it has been shown that higher motivation could induce greater P3 in delay discounting^83,84^, and previous fMRI studies showed that reward-related brain regions such as the accumbens showed larger activation in children than in adults^69^. Hence, children’s larger P3 responses during delay discounting in the current task might be related to their higher sensitivity and motivation to rewards, which is consistent with the finding that children exhibited higher sensitivity to rewards related to adults^69^. Third, children might view future rewards as an event with a lower possibility of occurrence since they have less experience with long time delays. From an evolutionary perspective, future rewards have been regarded as uncertain^85^. Furthermore, a previous study revealed that a lower probability of an infrequent target led to increased P3 amplitudes^86^. From these findings, it can be concluded that children might consider future rewards to be uncertain, which influences their decision-making process in delay discounting, while adults may be less influenced by the uncertainty of future rewards. More researches should be done to test the hypothesis.

Meanwhile, in other researches related to age differences in P3 components, the influences of the components occurring after P3 are often considered^53^. In our study, the possible potentials that might affect the P3 component are CNV (contingent negative variations), which was first described by Walter et al.^87^, and slow cortical potentials, which are often measured from 700 to 2200 ms after the stimulus onset in delay discounting task^88^. The two subcomponents of CNV are the O-wave and E-wave. The O-wave is an earlier occurring wave peaking about 700–1000 ms after the warning stimulus, which is involved in orientation to the warning stimulus and the E-wave is a later wave, which is linked to the expectation or preparation of responding^89,90^. Previous study found that children’s O-wave was smaller and occurred later than that of adults in parietal area due to developmental immaturity^53^. It seems that O-wave and low cortical potentials may affect the differences of P3 components between children and adults. However, from the grand average ERPs of children and adults in the present study, no such kind of obvious components were found. To further verify that differences of the components occurring after P3 may not affect the age differences of P3, we compared the mean amplitudes of children and adults from 650 to 950 ms, which was identified according to previous literatures^89,90^ and the grand average ERPs of all the conditions. No significant difference was observed between children and adults in this possible component by independent-samples t test (t (104) = 1.27, *p* = 0.21). Thus, it can be concluded that the found EEG effects of P3 were not attributed to potentials occurring after P3 such as CNV or slow cortical potentials but due to the developmental differences.

There were no significant correlations between ERP components and behaviors in children or adults, which is inconsistent with previous findings^19,46^. However, in our adult group, the correlation between N2 amplitudes and ratio of immediate choices was marginally significant. The difference between our study and others might be due to the different reward amount used in the researches. In Gui’s study^19^, the reward amount was larger than ours, which might be more attractive to participants. Thus, more cognitive control resources might be needed in their study. Meanwhile, for the whole sample of children and adults, although the behaviors were correlated significantly with P2 latencies, P3 amplitudes and latencies. However, according to the scatter-plots, we found that the correlations were spurious because of age-related mean differences. It is an interesting story whether the individuals that discounted similar in children and adults will show similar or different brain activities. As the sample was relatively small in our study, there was insufficient data to answer this question. Future researches should be done by using larger samples to find enough children and adults that discounted similarly to resolve this problem.

### Hemisphere effect on age

The hemisphere dominance of age on the task was reflected by children’s smaller P2 amplitudes on the right areas and smaller N2 amplitudes on the right and middle areas compared with adults, which showed that mainly the right side developed more slowly than the left side in delay discounting. The current age-related differences were over middle and right frontal areas but not left areas, which is similar to that reported in other studies. Stanger et al.^80^ found that the activation of right frontal-parietal areas correlated more with ln(k) values than the activation of left frontal-parietal areas, which indicated that individuals exhibiting higher degrees of discounting showed a larger right hemisphere effect. Additionally, the inhibition control process induced stronger activation over the right and middle areas of the frontal lobe, insula and parietal lobe in the response inhibition task^91^. What’s more, it was discovered that left hemisphere mature earlier than the corresponding regions of the right hemisphere and believed to be the reason that right-handed children with a left-dominant hemisphere might result in earlier maturation of left side and later maturation of the right side^49^. In our study, all the children were right-handed and the results sound reasonable. Therefore, it is plausible that the age differences in the delay discounting task, involving cognitive control, are exhibited mainly in the right hemisphere, which is the result of the activation of the two systems affected by age and the nature of the task. More fMRI studies should be performed to further test this hypothesis.

### Influences of reward amount and time delay on age

In the current study, the ratio of immediate choices was higher in large amount trials than in small amount trials, and it was higher in long delay trials than in short delay trials. According to our study design, a change in the reward amount occurred in the immediate options; hence, while a change in the time delay occurred in the delay options; it is understandable that individuals would make immediate choices more when the immediate amount was large or the delay time was long. Besides, children spent longer time making a choice in the large amount condition than in the small amount condition, while adults had similar RTs in both the small and large amount conditions, which showed an age effect of reward amount.

In the “bottom-up” stage reflected by P2 component, age difference was mainly in the small amount and long delay condition, with children exhibited smaller P2 amplitudes than adults. The P2 component is involved in early feature encoding on stimuli and attention filtering in the early decision-making period^35,46,80^. Smaller P2 amplitudes for children in the small amount and long delay condition indicated less effective of attention filtering than adults in early stage. Besides, it also showed that children paid less attention in small amount and long delay condition.

In the “top-down” stage related to cognitive control reflected by N2 responses, male children exhibited smaller N2 amplitudes in the small amount condition, and children showed longer N2 latencies in the long delay condition than in the short delay condition. Previous study found that N2 amplitude was negatively correlated with the ratio of immediate choices^19^, which suggested that smaller N2 amplitudes was correlated with impulsive behavior. In our study, male children’s smaller N2 amplitudes in small amount condition might exhibited that they recruited less cognitive control effort when rewards were small. Besides, children’s longer latencies in long delay condition indicated less effective of cognitive resources recruited and slower neural response speed in the stage.

When it comes to P3 response, another “top-down” component, related to cognitive processes, processing capacity and mental workload^45,66^. Children showed smaller amplitudes in small amount and long delay condition compared with the small amount and short delay condition and the large amount and long delay condition. However, adults showed no differences across conditions. Actually, choices in the small amount and short delay condition were more difficult than those in the small amount and long delay condition. Since the prior choices consisted of one unfavorable option in the amount dimension and a favored option in the time dimension; the latter choices consisted of unfavorable options in both amount and time dimensions. It can be concluded that for children, more difficult choices evoked larger P3 amplitudes and easier choices evoked smaller P3 amplitudes. It is possible that children could employ more neural resources to compute or evaluate the reward for more difficult choices than for easier choices. The results may also be explained by the motivational significance of children for the more difficult choices, as in the evaluation period, P3 amplitudes may also be influenced by motivation^45^, with more motivated stimuli evoking larger P3 amplitudes^92^.

From the components of P2, N2 and P3, we can find out that the age immaturities were mainly on responses in small amount and /or long delay condition, which may be due to the reason that children viewed rewards in this condition less valuable than adults. Children’s time sensitivity is immature^76^, which may lead to the result that they are more prone to be motivated by reward immediacy^1^. Besides, the rewards were relatively small in this condition. This may be the reason why children view rewards in small amount and/or long delay condition less valuable. Future researches could focus on the brain structure of different valuation pattern of children and adults in different reward conditions.

## Limitation

Apart from the findings in the present study, the following limitations must be considered for the future studies. First, in delay discounting, one should consider the reward amount and time delay at the same time, which may be correlated to one’s computation ability that is part of the general intelligence. Future research should collect one’s math performance meanwhile to see whether the age-related differences in delay discounting can be resulted from computation ability. Second, though our samples of children and adults were in the same area, their socio-economic status (SES) might be different. The children were all in the same elementary school and the adults were undergraduates or postgraduates. While previous researches showed that participants’ SESs and education degree were related to delay discounting^93–95^, future studies should consider these factors more carefully. Third, for the same amount of money children and adults may have different subjective values, which may influence their decision making in the task. Though some researchers found that reward valuation did not influence age differences in delay discounting by assessing the enjoyable degree when receiving the monetary rewards^62^, more methods should be used to test the impact of reward valuation on delay discounting such as testing one’s brain or skin electricity change. Fourth, as we discussed above, children’s different performance in delay discounting might be related to different time perception. More researches should be done to find out whether there is a causal correlation between time perception and delay discounting in the difference of children and adults.

## Conclusion

The current study found that children discounted monetary rewards more steeply than adults and more likely chose immediate choices. Regarding neural dynamic processes, children showed longer early detection and identification processing for the presented choices than adults. They also showed less mature cognitive control processing over frontal areas and devoted more neural efforts to make choices during the decision phase. Meanwhile, the age differences on neural activity were mainly in the right hemisphere in delay discounting. Moreover, the influences of reward amount and time delay on age was mainly represented during the neural responses in the small amount and/or long delay condition. The present study sheds light on the neural development of delay discounting in children and adults.

## Supplementary Information

Supplementary information.
