# Supplementary material for: The neurodevelopment of delay discounting for monetary rewards in pre-adolescent children
Source: Sci Rep. 2021 Apr 16;11:8337. doi: 10.1038/s41598-021-87282-z (PMC8052366; doi:10.1038/s41598-021-87282-z)
Supplement: Supplementary file 1 — Supplementary information. [file 41598_2021_87282_MOESM1_ESM.docx]

**The neurodevelopment of delay discounting for monetary rewards in pre-adolescent children**

**Mei Yu^1, 2^, Tongran Liu^1, 2*^, Fangfang Shangguan^3^, Jingxin Sui^4^, Jiannong Shi^1,2,5^**

1. *CAS Key Laboratory of Behavioral Science, Institute of Psychology, Chinese Academy of Sciences, Beijing 100101, China*
2. *Department of Psychology, University of Chinese Academy of Sciences, Beijing 100049, China*
3. *Beijing Key Laboratory of Learning and Cognition, School of Psychology, Capital Normal University, Beijing, China*
4. *Student Office, Liaoning Normal University, Dalian, 116029,Liaoning*

*5. Department of Learning and Philosophy, Aalborg University, Denmark*

**Corresponding author: Tongran Liu**

Address: CAS Key Laboratory of Behavioral Science, Institute of Psychology, Chinese Academy of Sciences, 16 Lincui Road, Chaoyang District, 100101 Beijing, China. Tel.: +86-10 64854533. Fax: +86-10-64872070. E-mail address: liutr@psych.ac.cn

**Appendix**

The hyperbolic model from Mazur**^1^** is written as:

V =A/(1+ kD)

where V denotes the subjective value of an outcome, A denotes its objective value, D denotes the delay to its receipt, and k denotes the rate of discounting**^1^**. The discounting rate k means the rate at which the subjective value of a reward decreases in value with respect to the time it takes to receive it**^2^**.

**Reference**

1. Mazur, J.E. An adjusting procedure for studying delayed reinforcement. In Commons, M.L., Mazur, J.E., Nevin, J.A., and Rachlin H. Eds., *The effect of delay and intervening events on reinforcement value, Quantitative Analysis of Behavior, Erlbaum, Hillsdale, New Jersey*, 55–73 (1987).

2. Myerson, J. & Green, L. Discounting of delayed rewards: Models of individual choice. *J. Exp. Anal. Behav.* **64**, 263–276 (1995).
